# An experienced physiotherapist prescribing and administering corticosteroid and local anaesthetic injections to the shoulder in an Australian orthopaedic service, a non-inferiority randomised controlled trial and economic analysis: study protocol for a randomised controlled trial

**DOI:** 10.1186/1745-6215-15-503

**Published:** 2014-12-21

**Authors:** Darryn Marks, Leanne Bisset, Michael Thomas, Shaun O’Leary, Tracy Comans, Shu Kay Ng, Philip G Conaghan, Paul Scuffham

**Affiliations:** Gold Cost Hospital and Health Service, Gold Coast University Hospital, 1 Hospital Boulevard, Southport, 4215 Gold Coast Australia; School of Allied Health Sciences, Griffith Health Institute, Griffith University, Gold Coast Campus, Kragujevac, Queensland 4222 Australia; NHMRC CCRE (Spinal Pain, Injury and Health), The University of Queensland, Brisbane, 4072 Australia; The Physiotherapy Department, Royal Brisbane and Women’s Hospital, Butterfield Street, Herston, Queensland 4029 Australia; School of Medicine, Griffith Health Institute, Griffith University, Logan Campus, University Drive, Meadowbrook, Queensland 4131 Australia; Leeds Institute of Rheumatic & Musculoskeletal Medicine, University of Leeds, & NIHR Leeds Musculoskeletal Biomedical Research Unit, Chapel Allerton Hospital, Leeds, LS7 4SA UK

**Keywords:** Extended scope practice, Physiotherapy, Physical therapy, Orthopaedic, Shoulder, Corticosteroid injection, Economic

## Abstract

**Background:**

The early management of orthopaedic outpatients by physiotherapists may be useful in reducing public hospital waiting lists. Physiotherapists in Australia are prevented by legislation and funding models from investigating, prescribing, injecting and referring autonomously. This gap in service is particularly noticeable in the management of shoulder pain in early-access physiotherapy services, as patients needing corticosteroid injection face delays or transfer to other services for this procedure. This trial will investigate the clinical (decision making and outcomes) and economic feasibility of a physiotherapist prescribing and delivering corticosteroid and local anaesthetic injections for shoulder pain in an Australian public hospital setting.

**Methods/Design:**

A double-blinded (patient and assessor) non-inferiority randomised controlled trial will compare an orthopaedic surgeon and a physiotherapist prescribing and delivering corticosteroid injections to the shoulder. Agreement in decision making between the two clinicians will be investigated, and economic information will be obtained for estimating disease burden and an economic evaluation. The surgeon and the physiotherapist will independently assess patients, and 64 eligible participants will be randomised to receive subacromial injection of corticosteroid and local anaesthetic from either the surgeon or the physiotherapist. Post-injection, all participants will receive physiotherapy. The primary outcome measure will be the Shoulder Pain and Disability Index measured at baseline, and at 6 and 12 weeks post-injection. Analysis will be conducted on an intention-to-treat basis and compared to a per-protocol analysis. A cost-utility analysis will be undertaken from the perspective of the health funder.

**Discussion:**

Findings will assist policy makers and services in improving access for orthopaedic patients.

**Trial registration:**

Australia and New Zealand Clinical Trials Registry: 12612000532808 First registered: 21 May 2012. First participant randomized: 16 January 2013.

## Background

Patients referred to Australian public health orthopaedic services face lengthy waiting periods. Publicly accessible information on outpatient waiting times is scant and inconsistent. The Australian National Outpatient Care Database does not report waiting times [1]. Queensland Health hospital performance data indicate that 54% of non-urgent outpatient referrals (not specifically orthopaedics) will take over a year to be seen [2], and data from some hospitals reveal that the waiting time to see orthopaedic specialists may extend beyond 3 years [3]. Furthermore, demands on orthopaedic services and subsequent waiting times are expected to worsen as the population ages [4] and rates of obesity and arthritis increase [5].

To combat future escalating delays for patients to access services such as orthopaedics, it has been proposed that the future health workforce should include greater role substitution and skill transference across professions [6]. The early management of orthopaedic referrals by physiotherapists is one example. Physiotherapists have demonstrated high levels of agreement with orthopaedic surgeons in the diagnosis and management of orthopaedic conditions of the hip, knee [7], and shoulder [8]. Orthopaedic models of care involving extended scope physiotherapy practice have also reported reduced costs, reduced waiting times and improved health outcomes; however, the quality of this evidence to date is generally low [9]. Corticosteroid injection by specially trained physiotherapists has been formally available in the UK since 2000 with a good safety record [10]. Contemporary UK laws and service delivery models permit appropriately trained physiotherapists autonomy in areas of prescribing, injecting, investigation and referral. In contrast, physiotherapists in Australia are prevented by legislation from prescribing and administering medicines. They are also hindered in requesting radiologist injection or referring to other specialties by Medicare funding rules, which require that the request be made by a doctor. While early physiotherapist assessment of orthopaedic patients has been implemented in some Australian hospitals, patients in need of specific pharmaceuticals or invasive procedures such as injection are typically returned to waiting lists because their needs are beyond the scope of Australian physiotherapists. This presents a service gap in comparison to UK models of care, particularly with regards to some orthopaedic conditions such as subacromial impingement syndrome of the shoulder.

Shoulder pain is a common problem, with a population prevalence of 20 to 33% [11], annual prevalence of 2.4% and an annual general practitioner (GP) care-seeking incidence of 1.5% [12]. Subacromial impingement syndrome encompasses multifactorial pathology and is the most frequent cause of shoulder pain [13]. Injection of corticosteroid and local anaesthetic to the subacromial space, sometimes combined with physiotherapy, is a common management strategy supported by clinical [14–16] and economic evidence [17–19]. The management of shoulder pain by Australian GPs is reported to be highly variable, often suboptimal and characterised by a high reliance upon specialist referral [20]. Yet, despite shoulder pain being one of the most frequent orthopaedic conditions referred to physiotherapists working in early-access orthopaedic services in Australia [21], these physiotherapists are unable to provide corticosteroid injection, resulting in delayed care for these patients. The capacity for injection delivery by physiotherapists in Australia may provide a solution; however, little is known of the clinical and economic outcomes of this model of care.

Existing literature concerning corticosteroid injection by physiotherapists is sparse and generally low quality. Positive health outcomes and the absence of adverse events have been reported; however, data are greatly limited to observational reports [22–24]. No clinical trials have yet directly investigated corticosteroid injection by physiotherapists. Some information pertaining to corticosteroid injection by physiotherapists may be inferred from studies into physiotherapist-led orthopaedic models of care that have included physiotherapist injection amongst treatments provided. One unblinded randomised clinical trial utilised superiority statistics and reported that partially supervised physiotherapist care was not significantly different to that of junior orthopaedic doctors in terms of health outcomes or patient satisfaction, but hospital costs were lower because physiotherapists requested fewer X-rays. In this study, some participants in the physiotherapy group received corticosteroid injection but the study did not report the body region injected, nor whether the physiotherapist actually delivered the injection [25]. Another unblinded (and non-randomised) clinical trial compared physiotherapist management of selected orthopaedic referrals to that of junior doctors and included physiotherapist corticosteroid injection as a treatment option. However, satisfaction and health outcome measures were not described and, again, the region of the injection was not reported [26]. Physiotherapists delivered injections in a shoulder treatment trial [27] but the efficacy of the physiotherapist as the provider of the injection was not investigated.

Therefore, the aim of this trial is to investigate the clinical and economic feasibility of a physiotherapist undertaking patient selection, and prescribing and delivering subacromial corticosteroid and local anaesthetic injection to the shoulder in an Australian public hospital setting. The primary aim of the study is to compare the clinical effectiveness of subacromial injection for shoulder pain, as delivered by a physiotherapist versus a consultant orthopaedic surgeon. Secondary study aims are to assess the agreement between the physiotherapist and orthopaedic surgeon in patient assessment and selection for injection, to estimate the economic burden of shoulder pain for patients awaiting orthopaedic care, and to calculate the costs of physiotherapist versus orthopaedic surgeon delivery of injections for shoulder pain. In our opinion, this is a novel and innovative study in that these research questions have not previously been investigated in a specific orthopaedic condition such as shoulder pain, in comparison to a consultant orthopaedic surgeon, or with a non-inferiority design. Furthermore, physiotherapist injection has not been previously reported in an Australian orthopaedic setting.

## Methods

### Design

A double-blinded (patient and outcome assessor) non-inferiority randomised controlled trial design will be used to compare injection decision and delivery by an orthopaedic surgeon to a model in which the physiotherapist makes the injection decision and gives the injection. Clinical decision-making between the two clinicians will also be compared. Economic data will be obtained for calculation of disease burden and a within-trial economic evaluation.

### Setting, process, inclusion and exclusion criteria

The study will take place at the Gold Coast Hospital and Health Service, with one upper limb staff specialist orthopaedic surgeon and one experienced physiotherapist who has prior training (in the UK) and experience in injecting and prescribing. A research assistant will identify appropriate orthopaedic referrals, contact potential participants, distribute study information, co-ordinate appointments and gain informed consent from participants for each part of the research. All participants will receive verbal and written information about the risks and benefits of participation and told that they can change their mind or withdraw at any time. Data will be stored in locked cabinets and password-protected digital files. Throughout the trial these will only be accessible by the research assistant and investigator (LB). The principal investigator will be given access at trial completion.

#### Part One and entry criteria for Part Two

**Figure 1:**
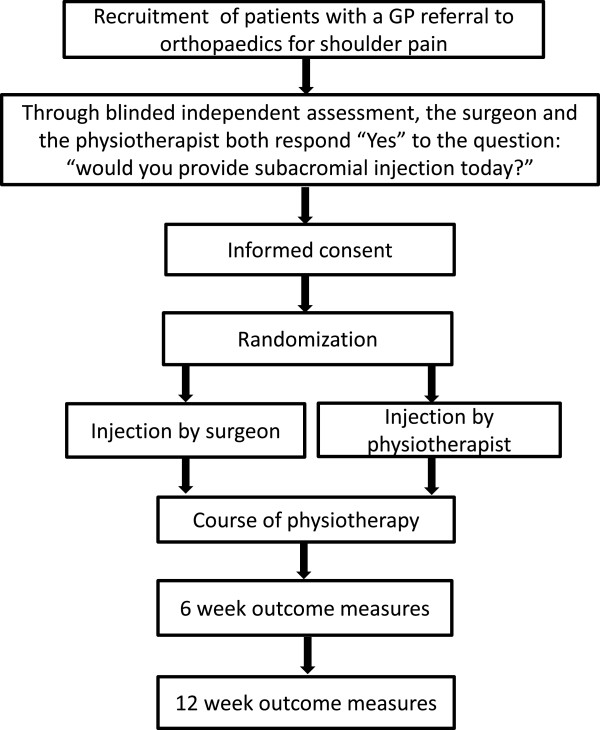
**Process of recruitment, consent, randomization, treatment and measurement.**

Participants will be included in Part One, the initial assessment phase of the study, if they are aged 18 years and over, have a new referral from their primary care doctor to the hospital orthopaedic department for shoulder pain and have the ability to read trial literature (in English) and give consent. Participants will be excluded if they have prior knowledge of either the research physiotherapist or the research orthopaedic surgeon (for example, from previous interactions or consultation), or have had no X-ray of the shoulder in the past 12 months. Written informed consent for Part One will be gained by the research assistant, following which baseline demographic and outcome measure data will be collected. The orthopaedic surgeon and physiotherapist, blind to the other’s assessment and decision, will independently examine each participant and their completed assessment forms will be compared by the research assistant. The order of examination (orthopaedic surgeon or physiotherapist first) will vary according to clinician availability. Participants will be unaware of decisions made by their first assessor while being examined by their second assessor. In bilateral presentations the side requested in the GP referral will be addressed. In bilateral referrals both sides will be assessed, the participant asked to nominate their most troublesome side and practitioners will make same-day injection decisions for one side only. Participants will be eligible to enter Part Two of the study if there is agreement between the orthopaedic surgeon and physiotherapist on the question “would you provide subacromial injection today?” In addition, Part Two participants must be able fill in the questionnaires and follow post-injection instructions. If the clinicians disagree about injection or the side to be injected or both mark “no”, the participant will take no further part in the research and receive care as directed by the surgeon. Additional exclusion criteria for entry into Part Two includes previous surgery to the involved shoulder, current use of anticoagulant medication, the need for prophylactic antibiotics with the injection, pregnancy or breastfeeding. The flow of participants into Part One and Part Two of the study are demonstrated in Figure 1.

#### Part Two, randomised controlled trial and randomisation process

Written informed consent for Part Two will be gained by the research assistant. Participants will then be randomised using concealed allocation to receive an injection from either the physiotherapist or the orthopaedic surgeon. The computer-generated randomisation sequence will be developed using Excel software by one investigator (LB) who will conceal the schedule from both the treating practitioners and the outcome assessor. Participants will be given a sealed opaque envelope by the research assistant, containing a standardised treatment sheet. This will direct the participant to one of two treatment rooms (Room A or Room B). The professional affiliation of the treating practitioner in each room will be unknown to both the study participants and the outcome assessor. Following participant randomisation to a treatment room (that is, a treating practitioner), the participant will provide written informed consent for the procedure before receiving the injection.

### Blinding

Throughout Part One the surgeon and the physiotherapist, blind to each other’s findings, will assess participants independently, in separate rooms. Participants will be blind to the profession of surgeon and the physiotherapist at all times throughout the entire trial. The outcome assessor will also be blind to the treating practitioner. Physiotherapists providing post-injection care will be asked to not seek information regarding the profession of the injecting clinician and not discuss the identity or profession of injecting clinicians with participants. They will be asked to report any breaches in blinding to the research assistant. The success of blinding will be assessed immediately post-injection, and at 6 and 12 weeks, when participants and the outcome assessor will each be asked to tick whether they think they received their injection from the physiotherapist or the surgeon. Unblinding of a participant will only be permitted in the event of an adverse event requiring management by the orthopaedic surgeon.

### Interventions

#### Injection delivered by surgeon or physiotherapist

Participants will receive, from either the physiotherapist or the surgeon, 1 ml betamethasone (Celestone Chronodose; 5.7 mg/ml) mixed with 5 ml 1% lignocaine hydrochloride, delivered to the subacromial space via an aseptic injection technique. Methylprednisolone acetate (1 ml; Depo-medrol; 40 mg/ml) will be used as an alternative, and recorded as such, if Celestone is unavailable. Standard post-injection advice will be given verbally and through a written information sheet advising reduced activity for 1 week, attendance at physiotherapy, as well as recognition of, and reporting procedures, for any adverse reactions.

#### Physiotherapy

All participants who were injected will be referred for a course of physiotherapy within the hospital’s outpatient physiotherapy department, beginning approximately 1 week post-injection. The injecting physiotherapist is not part of this department and will not provide post-injection treatment. Treating physiotherapists will be middle to senior outpatient staff with experience in treating musculoskeletal conditions. The treating physiotherapists will develop a consensus regarding best practice exercise and manual therapy interventions used for shoulder pain. Treatment will be delivered pragmatically with specific interventions and the number of sessions at the discretion of the treating physiotherapist. All treating physiotherapists will be directed to not deliver acupuncture to participants and this will be monitored by the research assistant.

### Outcome measures

Outcomes will be measured at baseline, and at 6 and 12 weeks follow-up by the blinded assessor. These time points were chosen on the basis of a previous study evaluating subacromial corticosteroid injection that found greater improvements in the injection group at 6 weeks than at 12 weeks [27]. At baseline the demographic, service, economic disease burden and clinical data will be recorded, including age, gender, duration of condition, imaging findings, dominant arm, affected side, medicines, occupation, shoulder-related direct and indirect costs, and usual service waiting time for care. Outcome measures and time points are displayed in Table 1.

**Table 1:** **Outcome measures and time points**

Outcome measure	Part of study		Time point (weeks)		
	Part one	Part two	0	6	12
Shoulder Pain and Disability Index	Yes	Yes	Yes	Yes	Yes
Impact and costs questionnaire	Yes		Yes		
Work Productivity and Activity Impairment Questionnaire	Yes		Yes		
Work Limitations Questionnaire	Yes		Yes		
Usual waiting time	Yes		Yes		
Examiner agreement	Yes		Yes		
European Quality of Life, five dimensions, five levels	Yes	Yes	Yes	Yes	Yes
Pain severity visual analogue scale	Yes	Yes	Yes	Yes	Yes
Range of movement/pain pre- and post-injection		Yes	Yes		
Adverse events		Yes	Yes, throughout		
Global perceived improvement		Yes	Yes	Yes	Yes
Satisfaction visual analogue scale		Yes			Yes
Post-injection physiotherapy		Yes			Yes
Labour costs		Yes			Yes
Medicine use	Yes	Yes			Yes

The table indicates which outcome measures will be used in each part of the trial and when each outcome measure is collected.

### Primary outcome measure

The primary outcome measure will be the Shoulder Pain and Disability Index (SPADI) measured at baseline, and at 6 and 12 weeks post-injection. The SPADI is a self-rating tool consisting of 13 questions with a scale of 0 to 10, divided into pain (five questions) and disability (eight questions) subscales. The two subscales are scored separately; an overall score is then calculated from the means of the two subscale scores, resulting in a total score varying from 0 (best) to 100 (worst) [28]. It has been widely used in shoulder research and has been shown to be reliable and valid, including use in patients with subacromial impingement syndrome of the shoulder [28, 29].

### Secondary outcome measures

#### Economic disease burden at baseline

An impact and costs questionnaire has been developed to gather shoulder-related direct and indirect costs including medical care, work earnings, impact and absence, and also costs of personal assistance required from others. A 3-month recall period was chosen based on previously reported patient accuracy in recall over this period [30, 31]. Impact on work productivity will be further assessed with two work productivity instruments which are validated in this patient population [32]. The Work Productivity and Activity Impairment Questionnaire has been used in many musculoskeletal conditions, is compatible with economic costing, and is easy to administer. The Work Limitations Questionnaire [33] is a widely used presenteeism measure with excellent construct validity and the benefit of being expressed as a percentage of productivity loss for dual clinical and economic analyses.

#### Usual waiting time for care

At the time of initial appointment for assessment, the usual waiting time to see the orthopaedic surgeon and the physiotherapist will be obtained from the hospital waiting list database.

#### Examiner agreement

The two examiners (physiotherapist and orthopaedic surgeon) will record their assessment, diagnosis, injection safety and management findings on standardised assessment forms.

#### Health-related quality of life

The European Quality of Life (five dimensions, five levels; EQ-5D-5 L) will allow quality of life to be expressed as utility values varying from 0 to 1, with 1 representing perfect health. This widely used scale builds upon the earlier EQ-5D-3 L [34] version.

#### Pain severity

A 100 mm visual analogue scale (VAS) anchored by “no pain” (0 mm) to worst pain imaginable (100 mm) will be used to rate participants “worst pain over the last 3 days”. The VAS has established validity and reliability, with extensive use in adults [35] and shoulder disorders [36].

#### Range of movement and pain immediately pre- and post-injection

Within a period of 20 minutes before and 20 minutes after injection, the blinded outcome assessor will measure shoulder range of movement three times in the scapular plane with a goniometer. The instruction will be to “take your arm up as far as you feel able”. Following the three measurement trials the participant will then rate on a 100 mm VAS anchored by “no pain” (0 mm) and worst pain imaginable” (100 mm) the severity of their worst pain and their mean pain during these three trials.

#### Adverse events

Participants will be instructed via the standardised post-injection information sheet to report any unwanted side-effects or adverse events to the research team. All adverse events will be recorded. A serious adverse event will be defined as one that requires hospitalization, results in persistent incapacity, or requires antibiotic treatment for infection, related to the injection, or anything worse.

#### Global perceived improvement

We will use a five-point Global Rating of Change scale, varying from completely recovered to much worse, to capture the participants impression of overall change in their shoulder condition. The Global Rating of Change has commonly been used for this purpose in painful shoulder conditions [37].

#### Satisfaction

Participants will be asked to rate their satisfaction with care over the 12-week period. A 100 mm VAS anchored by “not satisfied at all” (0 mm) to “completely satisfied” (100 mm) will be used.

#### Post-injection physiotherapy

The number of physiotherapy sessions provided for post-injection participants will be obtained from hospital booking systems.

#### Costs

Labour costs will be obtained from standard health service wage scales [38] for the assessing/injecting physiotherapist, the orthopaedic surgeon, and post-injection physiotherapy care. Participant medicine use will be recorded by the blinded assessor and costs taken from the Pharmaceutical Benefits Scheme database [39].

### Governance

#### Approvals

Ethical approval (Protocol version 4) and Site Specific Approval has been obtained through the Gold Coast Hospital and Health Service Human Research Ethics Committee, NHMRC code EC00160 (HREC/12/QGC/30; SSA/12/QGC/97), and Griffith University Human Research Ethics Committee (MED/23/13/HREC).

The research design requires that the physiotherapist act beyond the scope of usual professional practice in Australia and consequently a number of checks and approvals were required. These included legislative Approval under Queensland’s Health (Drugs and Poison) Regulation 1996, credentialing of overseas prescribing and injecting training against equivalent Australian standards, credentialing under Queensland’s Allied Health Framework for New/Complex practice, in addition to local hospital approvals.

#### Data and adverse event management

Adverse events reported by participants will be brought to the attention of the orthopaedic surgeon for clinical management as deemed appropriate. A Data Safety Monitoring Committee has been established comprising health service clinicians and experienced university academics, none of whom are involved in the study. Serious adverse events will be reported directly to both the Data Safety Monitoring Committee and the Human Research Ethics Committee. The biostatistician (SKN) will monitor the progress of the trial through interim statistical analyses at approximately one- and two-thirds completion. If the number of interim analyses is not small (for example, over five), the overall type I error rate could be controlled by adopting a higher level of significance for each interim statistical test [40]. Alternatively, a more formal alpha spending function approach [41] could be used to allocate some of the pre-specified overall type I error to each interim analysis. The interim analyses will be made available in confidence to the Data Safety Monitoring Committee and the Human Research Ethics Committee. The Data Safety Monitoring Committee will have authority to recommend trial cessation to the Sponsor.

#### Dissemination

Each participant will be mailed a plain language summary of the research results. Results will be also disseminated through publication, professional networks, meetings and conferences.

### Sample size

Assuming α = 0.05, β = 0.2, and a standard deviation of 21.7 point change in SPADI scores from baseline, we estimate that 54 participants (27 per group) will be required to test for comparative efficacy between the two treatments with a non-inferiority margin of 15 points in SPADI scores. This margin is specified based on clinical judgment and is approximately two-thirds of the minimum clinically important difference of 23.1 points for the SPADI at 6 weeks [29]. The standard deviation is obtained by adding 25% to the reported standard deviation for the change in SPADI scores at 6 weeks from baseline in a previous study comparing the responsiveness for SPADI and several indices in patients with shoulder pain receiving corticosteroid injection [42]. The power calculation is conservative in the sense that it ignores the added power inherent in the repeated measures design (assuming a first-order autoregressive covariance structure with a within-subject correlation coefficient of 0.5, the same sample size will have an increased power of above 90% using a generalised estimating equation (GEE) approach [43] for testing the difference in changes in scores between groups with two repeated follow-up measurements [44]). However, it is appropriate when treatment comparisons at the two individual time points (6 weeks and 12 weeks) are performed to assess the interaction treatment effect at different time after the injection. Allowing for a drop-out rate of 15%, we will recruit a total of 64 participants (32 per group) for injection. We anticipate this will require us to assess approximately 256 participants in total, because we estimate that 25% of participants assessed will be randomised for injection. This estimate is based on a study in which an orthopaedic surgeon sent 38% of patients for subacromial injection [8]. We anticipate some practitioner disagreement will bring the final figure to approximately 25%.

### Data analysis

All statistical analyses will be performed on an intention-to-treat basis with alpha level set at 0.05. As suggested in an extension of the CONSORT statement for non-inferiority trial design [45, 46], per-protocol analysis will be conducted and the results obtained by the two approaches will be compared [47].

#### Decision making

Agreement between the baseline assessment decisions from the two clinicians will be investigated using the proportion of observed agreement, the probability of chance agreement, the kappa statistics, and the Agreement Coefficient AC1 [48] with the 95% confidence intervals.

#### Clinical efficacy

For continuous outcomes, normality of data will be assessed and parametric tests applied if normality is upheld. Treatments from the two clinicians will be compared over time (pre- to post-intervention) on an intention-to-treat basis using the GEE [43] with a first-order autoregressive working correlation structure to account for within-subject correlation for repeated measurements. Robust estimator for covariance matrix will be adopted. The effects of time (within-group differences), practitioner (between-group differences), and practitioner by time interaction (between-group differences over time) will be included in all models and assessed using the Wald chi-square test. Non-inferiority of injection by physiotherapist to injection by surgeon will be declared if the upper limit of the one-sided confidence interval for the difference in mean change of SPADI in the physiotherapist group relative to the surgeon group is smaller than the non-inferiority margin of 15 points. Model fit and assumptions will be checked where appropriate within the GEE framework. The GEE approach works well with missing observations on outcome measures provided that they are missing completely at random [49], which will be tested using likelihood ratio statistics [50]. If the validity of the missing completely at random assumption was violated, adjustment using weighted GEE [51] will be performed. Appropriate *post hoc* tests will be conducted if significant main or interaction effects are identified from the omnibus analyses [52].

#### Economic evaluation

A within-trial economic evaluation, adhering to current guidelines [53], will be undertaken from the perspective of the health funder. A cost-utility ratio will be constructed using the formula: cost-utility = (Cost_i – Cost_c) /(QALY_i – QALY_c), where: QALY = quality-adjusted life years calculated by mapping the EQ-5D-5 L utility score across time and calculating the area under the curve, i = intervention group for main effect analysis, and c = control group. The time horizon for this economic evaluation will be 12 weeks. Direct costs will include labour costs of the clinician providing the injection, post-injection physiotherapy and change in medicine usage. Univariate, multivariate and probabilistic sensitivity analyses will be conducted to confirm stability of results and adjust for uncertainty in clinical and economic data.

## Discussion

The findings of this study will provide further evidence informing policy makers, regulators, health professionals, and the public as to the feasibility and safety of appropriately trained physiotherapists in Australia in requesting prescribing and administering medicines. The model of care to be evaluated in this study has the potential to reduce waiting times and improve access to appropriate and expedited care for patients who presently face long orthopaedic waiting times for specialist orthopaedic services. We anticipate this will have subsequent effects in improving patient flow, which could ultimately benefit both surgical and non-surgical orthopaedic pathways. The results may also have application for the management of other musculoskeletal presentations.

A non-inferiority randomised controlled trial design was chosen as it is appropriate when comparing an alternative treatment with an established proven effective treatment for a condition, and it is hypothesised that the alternative will not be superior in efficacy to the established treatment. In this case the objective is to determine whether the alternative treatment (physiotherapist injection) is not inferior to the existing gold standard (orthopaedic surgeon injection). This is done by assessing whether the alternative treatment falls within the non-inferiority margin [54].

This study will be limited by its involvement of only one injecting physiotherapist. This is necessary because there is presently no regulatory framework, recognised training curriculum or accreditation process for Australian physiotherapists to prescribe or administer medicines by injection. The physiotherapist in this study obtained governance approvals on the basis of their overseas training and is not representative of other physiotherapists in Australia. If physiotherapist-led prescribing and injecting were to be implemented on a wider scale, appropriate training and regulatory frameworks would be required.

Further potential limitations of the study include differences in practice recency and frequency between the surgeon and the physiotherapist, as governance approvals permit the physiotherapist to inject only within the trial. In contrast the surgeon has recent practice and will have regular ongoing injection practice through their work outside of the research.

In summary, several knowledge gaps will be addressed by this research. It will evaluate the safety, feasibility, and cost of physiotherapists injecting in an Australian setting. To our knowledge, it will also, for the first time, compare the decision-making processes and clinical efficacy surrounding injection by a physiotherapist to that of a consultant orthopaedic surgeon, for a specific orthopaedic condition. We believe this to be the first study of a physiotherapist injecting corticosteroid in Australia, and it could serve as a benchmark from which subsequent studies can be conceived to further our understanding of the potential health benefits of extended-scope activity within physiotherapy, as well as other allied health professions.

## Trial status

Recruitment began in January 2013, with the first participant randomized 16 January 2013.

## Abbreviations

EQ-5D-5 L: European quality of life (five dimensions, five levels)
GEE: Generalised estimating equation
GP: General practitioner
SPADI: Shoulder Pain and Disability Index
VAS: Visual analogue scale.
